# A Case of Hepatocellular Carcinoma Associated with Hepatic Sarcoidosis

**DOI:** 10.31662/jmaj.2024-0387

**Published:** 2025-01-31

**Authors:** Yasuo Nagai, Kosei Takagi, Kazuya Yasui, Kazuya Kariyama, Tomokazu Fuji, Toshiyoshi Fujiwara

**Affiliations:** 1 Department of Gastroenterological Surgery, Okayama University Graduate School of Medicine, Dentistry, and Pharmaceutical Sciences, Okayama, Japan; 2 Department of Gastroenterology, Okayama City Hospital, Okayama, Japan

**Keywords:** hepatocellular carcinoma, hepatic sarcoidosis, peritoneal sarcoidosis

A 62-year-old patient with lung sarcoidosis presented with a 1.6-cm liver tumor. The patient reported no history of alcohol consumption or viral hepatitis. During the 3-year follow-up, the liver biopsies revealed hepatic sarcoidosis with no malignancy. However, the tumor eventually grew to 4.0 cm; radiographic findings are presented in Figure 1. The tumor was diagnosed as hepatocellular carcinoma (HCC). Consequently, robotic hepatectomy was performed, and intraoperative and pathological findings are demonstrated in Figure 2. Finally, the tumor was diagnosed as HCC associated with hepatic sarcoidosis.

**Figure 1.**
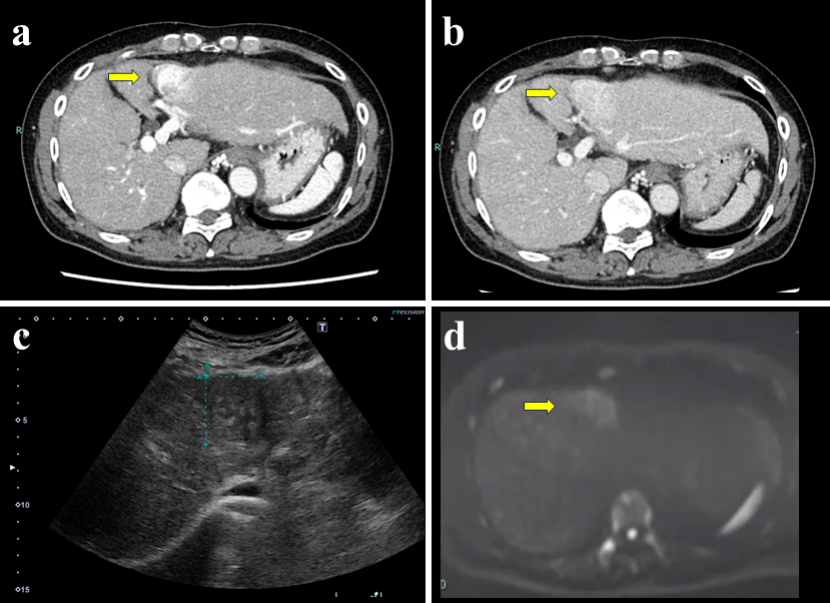
(a) Contrast-enhanced computed tomography images showing an early enhancement tumor at segment 3; the liver shape suggested a cirrhotic pattern. (b) Washout on delayed phase; (c) abdominal ultrasound showing mosaic architecture; (d) magnetic resonance imaging showing high signal intensity on diffusion-weighted images.

**Figure 2.**
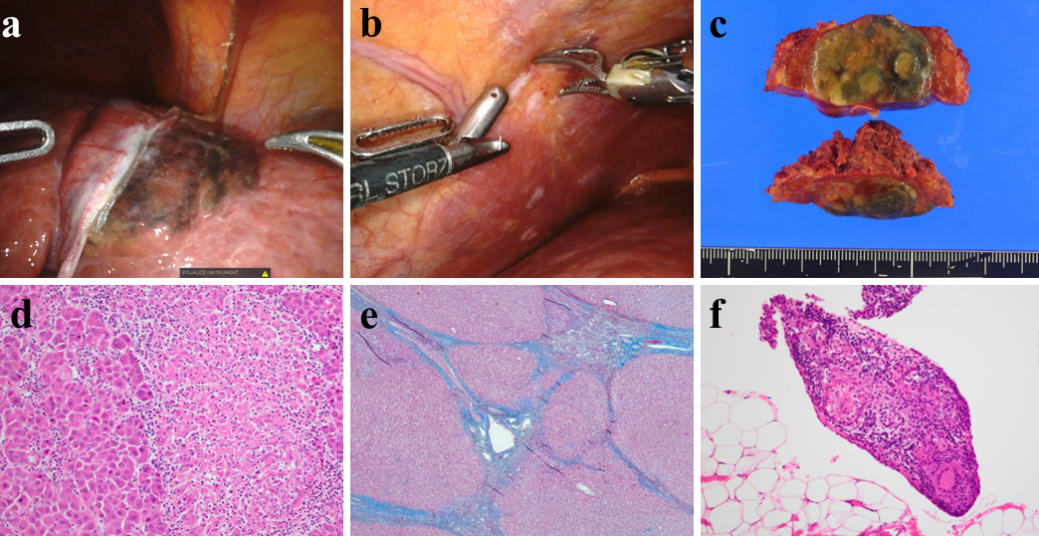
(a) Intraoperative findings showing a well-defined black color tumor on the segment 3 surface; (b) multiple white peritoneal nodules; (c) macroscopic findings showing a 3.2-cm green-yellowish tumor, capsulated confluent multinodular type; (d) microscopic findings showing moderately differentiated hepatocellular carcinoma and epithelioid granulomas; (e) background liver histology showing bridging fibrosis and regenerating nodules accompanied by the distortion of the liver lobules (f4); (f) epithelioid granulomas in a peritoneal nodule, suggesting peritoneal sarcoidosis.

Reportedly, the incidence of hepatic sarcoidosis is 4.2% in patients with sarcoidosis ^(1)^. Nonetheless, few studies have reported sarcoidosis associated with HCC ^(2), (3)^. Herein, the liver cirrhosis occurred because of hepatic sarcoidosis, further developing HCC. In summary, this report describes the clinical images of an extremely rare case of HCC associated with hepatic sarcoidosis.

## Article Information

### Conflicts of Interest

None

### Author Contributions

YN and KT wrote the draft. All authors contributed equally to the manuscript. All authors edited, read, and approved the final manuscript.

### Approval by Institutional Review Board (IRB)

Not applicable

### Informed Consent

Written informed consent was obtained from the patient for the publication of this report and any accompanying images.
